# Supplementary material for: Identification and Validation of Ferroptosis-Related DNA Methylation Signature for Predicting the Prognosis and Guiding the Treatment in Cutaneous Melanoma
Source: Int J Mol Sci. 2022 Dec 10;23(24):15677. doi: 10.3390/ijms232415677 (PMC9778758; doi:10.3390/ijms232415677)
Supplement: Supplementary file 1 [file ijms-23-15677-s001.zip › ijms-2038767-supplementary.pdf]

Identification and validation of ferroptosis-related DNA methylation signature for predicting the prognosis and guiding the treatment in cutaneous melanoma

Wenna Guo[1], Xue Wang[1], Yanna Wang[1], Shuting Zhu[1], Rui Zhu[2*], Liuun Zhu[2*]

Table S1. The top 50 Gene Ontology (GO) terms of differentially up-regulated genes in low-risk group.

GO Term	ID	Input number	Background number	Enrichment ratio	*p*-Value	Corrected *p*-Value
protein binding	GO:0005515	389	11779	0.033	2.22E-85	4.02E-82
plasma membrane	GO:0005886	238	4619	0.052	1.21E-75	1.46E-72
immune response	GO:0006955	85	427	0.199	2.15E-65	1.56E-62
innate immune response	GO:0045087	88	558	0.158	3.86E-60	2.00E-57
external side of plasma membrane	GO:0009897	77	392	0.196	8.57E-59	3.88E-56
adaptive immune response	GO:0002250	61	347	0.176	4.45E-44	1.34E-41
inflammatory response	GO:0006954	62	381	0.163	5.02E-43	1.40E-40
extracellular region	GO:0005576	116	1843	0.063	2.77E-41	7.17E-39
regulation of immune response	GO:0050776	47	221	0.213	2.06E-37	4.67E-35
cytoplasm	GO:0005737	177	4624	0.038	3.18E-36	6.78E-34
integral component of plasma membrane	GO:0005887	92	1380	0.067	2.77E-34	5.57E-32
cytokine-mediated signaling pathway	GO:0019221	47	282	0.167	3.41E-33	6.18E-31

extracellular exosome	GO:0070062	108	2085	0.052	2.80E-31	4.61E-29
extracellular space	GO:0005615	92	1572	0.059	3.18E-30	5.01E-28
defense response to virus	GO:0051607	39	200	0.195	5.71E-30	8.62E-28
cell surface receptor signaling pathway	GO:0007166	43	286	0.150	8.07E-29	1.12E-26
cytosol	GO:0005829	169	5095	0.033	3.25E-27	4.37E-25
transmembrane signaling receptor activity	GO:0004888	32	146	0.219	3.57E-26	4.32E-24
B cell receptor signaling pathway	GO:0050853	30	122	0.246	7.08E-26	8.28E-24
identical protein binding	GO:0042802	81	1456	0.056	3.17E-25	3.59E-23
response to virus	GO:0009615	28	104	0.269	3.70E-25	4.07E-23
signal transduction	GO:0007165	67	1013	0.066	8.68E-25	9.25E-23
cell surface	GO:0009986	52	598	0.087	2.46E-24	2.55E-22
neutrophil degranulation	GO:0043312	47	482	0.098	5.32E-24	5.35E-22
signaling receptor binding	GO:0005102	40	353	0.113	9.48E-23	9.05E-21
cellular response to interferon-gamma	GO:0071346	25	95	0.263	2.15E-22	2.00E-20
membrane	GO:0016020	92	2075	0.044	5.13E-22	4.65E-20
type I interferon signaling pathway	GO:0060337	22	66	0.333	9.80E-22	8.46E-20
positive regulation of T cell proliferation	GO:0042102	21	61	0.344	4.77E-21	3.93E-19
phagocytosis, engulfment	GO:0006911	25	111	0.225	5.61E-21	4.52E-19
leukocyte migration	GO:0050900	30	192	0.156	6.91E-21	5.45E-19
interferon-gamma-mediated signaling pathway	GO:0060333	21	71	0.296	6.56E-20	4.89E-18
apoptotic process	GO:0006915	44	546	0.081	1.45E-19	1.03E-17
chemokine-mediated	GO:0070098	20	67	0.299	4.31E-19	2.95E-17

signaling pathway						
integral component of membrane	GO:0016021	121	3643	0.033	6.21E-19	4.17E-17
immunological synapse	GO:0001772	17	40	0.425	1.51E-18	9.62E-17
positive regulation of interferon-gamma production	GO:0032729	19	63	0.302	2.83E-18	1.77E-16
Fc-gamma receptor signaling pathway involved in phagocytosis	GO:0038096	24	135	0.178	4.14E-18	2.54E-16
complement activation, classical pathway	GO:0006958	25	156	0.160	7.42E-18	4.43E-16
cellular response to lipopolysaccharide	GO:0071222	25	158	0.158	9.72E-18	5.59E-16
positive regulation of ERK1 and ERK2 cascade	GO:0070374	27	207	0.130	3.84E-17	2.08E-15
chemotaxis	GO:0006935	22	119	0.185	4.99E-17	2.66E-15
neutrophil chemotaxis	GO:0030593	19	77	0.247	6.76E-17	3.55E-15
T cell receptor signaling pathway	GO:0050852	25	174	0.144	7.44E-17	3.85E-15
regulation of complement activation	GO:0030449	21	113	0.186	2.35E-16	1.17E-14
T cell costimulation	GO:0031295	16	53	0.302	1.32E-15	6.28E-14
T cell activation	GO:0042110	15	44	0.341	2.30E-15	1.07E-13
defense response to bacterium	GO:0042742	25	209	0.120	3.49E-15	1.58E-13
positive regulation of GTPase activity	GO:0043547	29	307	0.094	6.16E-15	2.72E-13
antigen binding	GO:0003823	21	139	0.151	9.25E-15	3.95E-13

Table S2. The top 50 KEGG pathway of differentially up-regulated genes in low-risk group.

**KEGG pathway**	**ID**	**Input number**	**Background number**	**Enrichment ratio**	***p*-Value**	**Corrected *p*-Value**
Cytokine-cytokine receptor interaction	hsa04060	48	294	0.163	1.62E-33	3.10E-31
Viral protein interaction with cytokine and cytokine receptor	hsa04061	29	100	0.29	8.63E-27	1.12E-24
Chemokine signaling pathway	hsa04062	35	190	0.184	2.79E-26	3.49E-24
Primary immunodeficiency	hsa05340	19	37	0.513	8.50E-22	7.51E-20
NF-kappa B signaling pathway	hsa04064	23	100	0.23	1.40E-19	1.01E-17
Cell adhesion molecules (CAMs)	hsa04514	26	146	0.178	1.59E-19	1.11E-17
B cell receptor signaling pathway	hsa04662	21	82	0.256	8.12E-19	5.35E-17
Osteoclast differentiation	hsa04380	24	128	0.187	1.41E-18	9.09E-17
Staphylococcus aureus infection	hsa05150	19	68	0.279	9.43E-18	5.51E-16
Hematopoietic cell lineage	hsa04640	21	97	0.216	1.57E-17	8.74E-16
Tuberculosis	hsa05152	25	179	0.139	1.35E-16	6.81E-15
Natural killer cell mediated cytotoxicity	hsa04650	21	131	0.160	3.23E-15	1.48E-13
Phagosome	hsa04145	22	152	0.145	4.72E-15	2.11E-13
T cell receptor signaling pathway	hsa04660	19	103	0.184	7.09E-15	3.10E-13
Leishmaniasis	hsa05140	17	74	0.230	8.09E-15	3.49E-13

Epstein-Barr virus infection	hsa05169	24	201	0.119	1.30E-14	5.34E-13
Complement and coagulation cascades	hsa04610	17	79	0.215	2.06E-14	8.30E-13
Leukocyte transendothelial migration	hsa04670	19	112	0.170	2.72E-14	1.06E-12
PD-L1 expression and PD-1 checkpoint pathway in cancer	hsa05235	17	89	0.191	1.14E-13	4.21E-12
Th17 cell differentiation	hsa04659	17	107	0.159	1.60E-12	5.21E-11
Influenza A	hsa05164	20	167	0.120	2.00E-12	6.30E-11
Pertussis	hsa05133	15	76	0.197	2.11E-12	6.57E-11
Rheumatoid arthritis	hsa05323	15	91	0.165	2.06E-11	5.67E-10
Measles	hsa05162	17	138	0.1235	6.10E-11	1.62E-09
Jak-STAT signaling pathway	hsa04630	18	162	0.112	7.85E-11	2.00E-09
Th1 and Th2 cell differentiation	hsa04658	14	92	0.152	2.48E-10	6.00E-09
NOD-like receptor signaling pathway	hsa04621	18	178	0.101	3.20E-10	7.47E-09
Malaria	hsa05144	11	49	0.224	5.70E-10	1.28E-08
Human immunodeficiency virus 1 infection	hsa05170	19	212	0.090	6.75E-10	1.46E-08
Toll-like receptor signaling pathway	hsa04620	14	104	0.135	1.05E-09	2.26E-08
Toxoplasmosis	hsa05145	14	113	0.124	2.80E-09	5.63E-08
Fc gamma R-mediated phagocytosis	hsa04666	13	94	0.138	3.10E-09	6.14E-08
Yersinia infection	hsa05135	14	121	0.116	6.23E-09	1.18E-07
Chagas disease (American trypanosomiasis)	hsa05142	13	103	0.126	8.41E-09	1.52E-07
Fc epsilon RI signaling pathway	hsa04664	11	68	0.162	1.19E-08	2.04E-07

Systemic lupus erythematosus	hsa05322	14	133	0.105	1.87E-08	3.08E-07
Pathways in cancer	hsa05200	27	530	0.051	2.32E-08	3.76E-07
Transcriptional misregulation in cancer	hsa05202	16	186	0.086	2.56E-08	4.03E-07
Regulation of actin cytoskeleton	hsa04810	17	214	0.079	2.80E-08	4.35E-07
Intestinal immune network for IgA production	hsa04672	9	49	0.184	9.76E-08	1.39E-06
TNF signaling pathway	hsa04668	12	112	0.107	1.63E-07	2.22E-06
Human T-cell leukemia virus 1 infection	hsa05166	16	219	0.073	2.08E-07	2.78E-06
Human cytomegalovirus infection	hsa05163	16	225	0.071	2.92E-07	3.78E-06
Cytosolic DNA-sensing pathway	hsa04623	9	63	0.143	6.58E-07	7.87E-06
PI3K-Akt signaling pathway	hsa04151	19	354	0.054	1.35E-06	1.53E-05
African trypanosomiasis	hsa05143	7	37	0.189	2.23E-06	2.40E-05
Legionellosis	hsa05134	8	55	0.145	2.45E-06	2.60E-05
Antigen processing and presentation	hsa04612	9	77	0.117	3.00E-06	3.14E-05
Inflammatory bowel disease (IBD)	hsa05321	8	65	0.123	7.54E-06	7.32E-05
Platelet activation	hsa04611	10	124	0.081	1.79E-05	1.56E-04

Table S3. The top 50 Reactome pathway of differentially up-regulated genes in low-risk group.

**Reactome pathway**	**ID**	**Input number**	**Background number**	**Enrichment ratio**	***p*-Value**	**Corrected *p*-Value**
Immune System	R-HSA-168256	227	2096	0.108	4.58E-133	1.66E-129
Cytokine Signaling in	R-HSA-1280215	107	836	0.128	3.34E-65	2.02E-62

Immune system						
Innate Immune System	R-HSA-168249	109	1043	0.105	2.49E-58	1.00E-55
Adaptive Immune System	R-HSA-1280218	90	748	0.120	1.57E-52	5.68E-50
Signaling by Interleukins	R-HSA-449147	69	619	0.111	2.91E-38	7.04E-36
Interferon Signaling	R-HSA-913531	41	194	0.211	1.15E-32	1.99E-30
Immunoregulatory interactions between a Lymphoid and a non-Lymphoid cell	R-HSA-198933	33	128	0.258	6.56E-29	9.51E-27
Neutrophil degranulation	R-HSA-6798695	46	478	0.096	2.70E-23	2.65E-21
Interferon alpha/beta signaling	R-HSA-909733	22	67	0.328	1.29E-21	1.08E-19
Interferon gamma signaling	R-HSA-877300	23	90	0.256	1.83E-20	1.41E-18
Hemostasis	R-HSA-109582	47	617	0.076	6.61E-20	4.89E-18
Interleukin-10 signaling	R-HSA-6783783	17	47	0.362	1.37E-17	7.75E-16
Chemokine receptors bind chemokines	R-HSA-380108	17	48	0.354	1.83E-17	1.00E-15
Interleukin-2 family signaling	R-HSA-451927	16	44	0.364	1.15E-16	5.89E-15
Cell surface interactions at the vascular wall	R-HSA-202733	22	135	0.163	5.21E-16	2.55E-14
Interleukin-4 and Interleukin-13 signaling	R-HSA-6785807	20	108	0.185	1.29E-15	6.23E-14
Signal Transduction	R-HSA-162582	93	2689	0.035	1.61E-15	7.58E-14

Class A/1 (Rhodopsin-like receptors)	R-HSA-373076	29	322	0.090	1.91E-14	7.76E-13
Antigen processing-Cross presentation	R-HSA-1236975	18	98	0.184	3.89E-14	1.50E-12
Antigen activates B Cell Receptor (BCR) leading to generation of second messengers	R-HSA-983695	12	31	0.387	4.30E-13	1.51E-11
Regulation of Complement cascade	R-HSA-977606	13	47	0.277	1.59E-12	5.21E-11
Interleukin-3, Interleukin-5 and GM-CSF signaling	R-HSA-512988	13	47	0.277	1.59E-12	5.21E-11
Peptide ligand-binding receptors	R-HSA-375276	21	188	0.112	1.89E-12	6.07E-11
Signaling by the B Cell Receptor (BCR)	R-HSA-983705	17	110	0.155	2.38E-12	7.25E-11
TCR signaling	R-HSA-202403	17	117	0.145	5.77E-12	1.70E-10
Fcgamma receptor (FCGR) dependent phagocytosis	R-HSA-2029480	15	83	0.181	6.44E-12	1.88E-10
Complement cascade	R-HSA-166658	13	57	0.228	1.29E-11	3.63E-10
GPCR ligand binding	R-HSA-500792	29	454	0.064	5.19E-11	1.39E-09
Class I MHC mediated antigen processing & presentation	R-HSA-983169	26	370	0.070	7.58E-11	1.95E-09
Regulation of actin dynamics for phagocytic cup formation	R-HSA-2029482	12	59	0.203	2.53E-10	6.07E-09

G alpha (i) signalling events	R-HSA-418594	26	405	0.064	4.71E-10	1.08E-08
Costimulation by the CD28 family	R-HSA-388841	12	65	0.185	6.74E-10	1.46E-08
FCGR activation	R-HSA-2029481	7	10	0.700	1.56E-09	3.21E-08
Platelet activation, signaling and aggregation	R-HSA-76002	20	260	0.077	2.81E-09	5.64E-08
Interleukin-2 signaling	R-HSA-9020558	7	12	0.583	3.95E-09	7.66E-08
Generation of second messenger molecules	R-HSA-202433	9	32	0.281	3.95E-09	7.66E-08
PD-1 signaling	R-HSA-389948	8	22	0.364	5.51E-09	1.05E-07
GPVI-mediated activation cascade	R-HSA-114604	9	35	0.257	7.71E-09	1.41E-07
Disease	R-HSA-1643685	41	1049	0.039	9.23E-09	1.64E-07
Fc epsilon receptor (FCERI) signaling	R-HSA-2454202	14	130	0.108	1.44E-08	2.44E-07
Interleukin receptor SHC signaling	R-HSA-912526	8	26	0.308	1.63E-08	2.73E-07
Other interleukin signaling	R-HSA-449836	19	275	0.069	3.51E-08	5.32E-07
FCERI mediated Ca+2 mobilization	R-HSA-2871809	8	30	0.267	4.18E-08	6.29E-07
ER-Phagosome pathway	R-HSA-1236974	11	82	0.134	6.74E-08	9.84E-07
CD22 mediated BCR regulation	R-HSA-5690714	5	5	1.000	1.16E-07	1.63E-06
Signal regulatory protein family interactions	R-HSA-391160	6	13	0.462	1.55E-07	2.12E-06

Classical antibody-mediated complement activation	R-HSA-173623	5	6	0.833	2.10E-07	2.80E-06
DAP12 interactions	R-HSA-2172127	8	39	0.205	2.41E-07	3.18E-06
The role of Nef in HIV-1 replication and disease pathogenesis	R-HSA-164952	7	26	0.269	2.83E-07	3.71E-06
Signaling by SCF-KIT	R-HSA-1433557	8	40	0.200	2.86E-07	3.71E-06

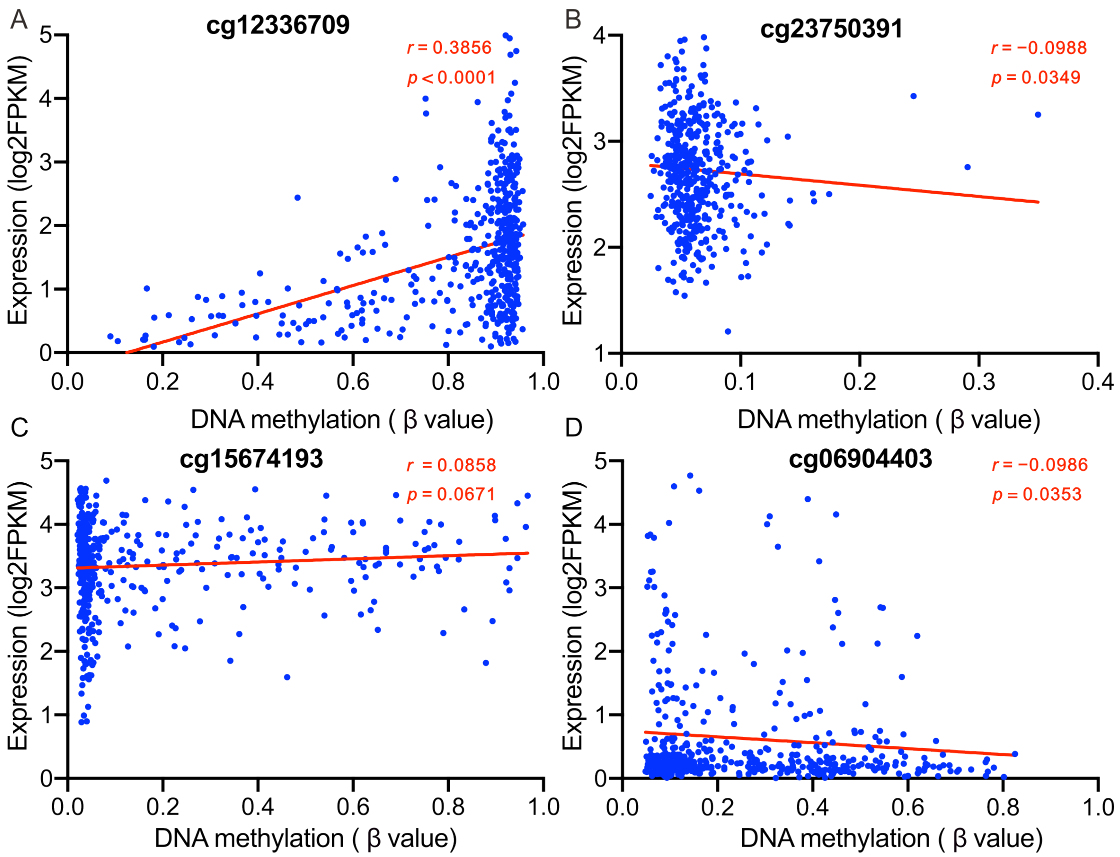

Figure S1. Relationship between methylation level and its gene expression level of each site was evaluated using Spearman correlation analysis. Gene expression levels are reported as log_2(FPKM), and methylation β-values are defined by the Infinium HumanMethylation 450 BeadChip. The Reported *p* values are bipartite.

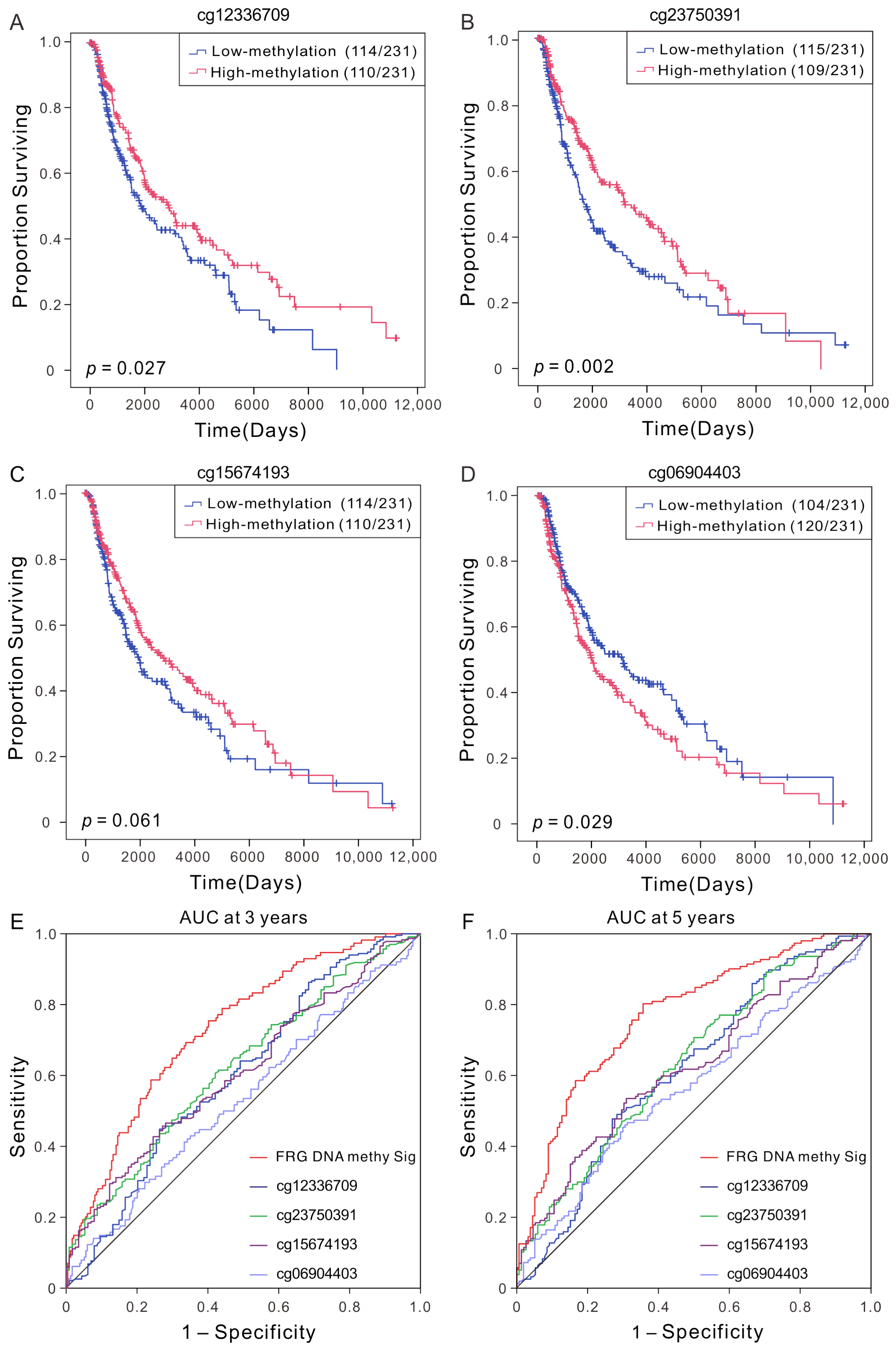

Figure S2. Association of single DNA methylation sites with patient OS. (A-D) Kaplan–Meier analysis with Wilcoxon test was performed to estimate the differences in OS between patients with different DNA methylation levels. **(E-F)** The AUC value of individual methylation level in predicting 3-year and 5-year OS.
